# The Influence of the Accelerated Aging Process on the Compressive Strength of Wood Treated with Components of a Salt Fire Retardant

**DOI:** 10.3390/ma15144931

**Published:** 2022-07-15

**Authors:** Wojciech Łukasz Grześkowiak, Marta Molińska-Glura, Marcelina Przybylska

**Affiliations:** 1 Department of Mechanical Wood Technology, Faculty of Forestry and Wood Technology, Poznan University of Life Sciences, Wojska Polskiego 28, 60-637 Poznan, Poland; marcelina.k.przybylska@gmail.com; 2 Department of Economics and Forest Technology, Faculty of Forestry and Wood Technology, Poznan University of Life Sciences, Wojska Polskiego 28, 60-637 Poznan, Poland; marta.glura@up.poznan.pl

**Keywords:** wood, fire retardants, artificial ageing, compression strength

## Abstract

This paper presents the results of research on the influence of the components of salt flame retardants on the compressive strength of wood depending on the time of accelerated aging. The effect of the agent was assessed on the basis of the change in the strength of treated wood compared to that of untreated wood. In addition, a statistical analysis of the obtained results was used to determine which of the components most significantly affect the changes in the compressive strength of wood along the fibers, and to what extent. It was found that extending the aging process time in the case of control and boric acid-protected samples did not significantly change the strength properties. It has also been found that some compounds contained in fire retardant have an antagonistic effect related to the compressive strength of wood.

## 1. Introduction

Fire is the fastest destructive factor for wood, but it is impossible to eliminate it in everyday life. Various measures to reduce the flammability of wood material have been used for centuries, but they are still incapable of completely preventing the burning of this material. They allow for a significant reduction of parameters, such as the spread of flames over the surface, weight loss, or the rate of heat release. Among the fire retardants, salt compounds and mixtures thereof are most commonly used. They include: phosphates, ammonium sulphates and their derivatives, boric acid, borax, urea, and potassium carbonate [1,2]. Undoubted advantages of fire-retardant treatment, after years of use, may have negative consequences for the structural strength of the protected wood. The strength properties are influenced, among others, by the type of flame retardant, its pH, impregnation technology and wood humidity, and the conditions in which the wooden elements are used. Publications presenting research on the fire retardants effectiveness of preparations clearly confirm the deterioration of wood strength on the basis of parameters such as: modulus of elasticity (E) and compressive strength (Rm) [1,3,4]. In the research by Grześkowiak et al. [5] on the fire-retardant use of potassium carbonate, it was found that the strength of pine wood, treated with a 20% solution, was decreased by 20% compared to unprotected wood. Moreover, aspen wood impregnated with the same solution showed a decrease in compressive strength by about 15% when impregnated with the vacuum-pressure method using. The research by Surmiński and Lutomski [6] on the effect of treatment of pine wood with fire retardant preparations also showed that wood treated with the vacuum-pressure method with 25% K_2_CO_3_ showed a decrease in compressive strength by 11.78%. When impregnating the wood with 25% NH_4_H_2_PO_4_, (NH_4_)_2_SO_4_ mixture using the vacuum method, an improvement in wood strength of 2% was found compared to unprotected wood. The same authors using 30% vacuum treatment with the NH_4_H_2_PO_4_, (NH_4_)_2_SO_4_, H_3_BO_3_, Na_2_B_4_O mixture achieved an improvement in compressive strength of 13.19%. The product [7], using a 10% mixture of phosphates, sulphates, boron compounds, and salts of benzoic acid, achieved a strength improvement of 7.1% compared to unprotected wood. The conducted research on long-term heating of wood protected with, among others: phosphoric acid (PA), ammonium dihydrogen phosphate (MAP) or a mixture of borax and boric acid (BBA) showed that phosphoric acid had the greatest impact on the decrease in strength. The mixture of borax and boric acid deteriorated the strength of the wood the least [8,9]. In tests made by Sweet et al. [9], three variants of temperature and humidity were used, imitating: the temperature in a dry room (27 °C), the temperature to which the roof sheathing may be exposed (54 °C), and finally 80 °C. Both during and after the exposure, measurements were made of the modulus of elasticity, moisture, modulus of breaking, and maximum load. It was found that the factor determining the strength drop was the nature of the chemical compound used, followed by the exposure conditions and the method of saturation. In research on the influence of the conditions of use on the properties of protected wood, simulations of these conditions are used, known as accelerated aging. Changes in wood resulting from accelerated, artificial aging should correspond to the changes that would be caused by exposure to weather conditions, with particular emphasis on the effects of humidity and temperature. Wood heated in the atmosphere of water vapor requires lower decomposition activation energy than raw material at elevated temperature, but in a dry environment [10,11,12,13]. There are many methods based on the simulation of natural conditions in the laboratory, differing in cycle duration, annealing and freezing temperatures. For example, the ASTM D 1037 method [14] provides a 60-h cycle consisting in soaking the wood, steam, freezing, steam and heating in dry air. Kajita et al. [15] give an accelerated aging method based on BS 5669-1:1989 [16] and consisting in immersion in water at 20 °C for 36 h, freezing at −12 °C for 24 h, and heating in dry air at 70 °C for 36 h. On the other hand, Riwier [17] provides two methods of accelerated aging of the wood used: Cyclic-boil dry method, which consists in repeatedly submerging the material in hot water and heating with dry air, and the vacuum-pressure-dry cycle method, which consists in immersing the wood in water under negative pressure, reducing vacuum, pressure and heating with dry air. As can be seen from the above description, the applied conditions of artificial aging differ in terms of values and duration of action, etc. Accelerated aging is purely theoretical, because it is impossible to transfer this long-term process to laboratory conditions, so as to accurately reproduce the changes taking place in the structure of the tested materials. 

In this study, it was decided to focus on determining the effect of fire-retardant treatment of wood and accelerated aging time on the strength parameters of the protected wood. It was decided to protect the wood with chemical compounds included in the fire-retardant preparation, as well as with the fire-retardant preparation itself, in order to determine which of the compounds most significantly influences the changes in the compressive strength of the wood along the fibers, and to what extent. Flammability properties of the tested commercial fire retardant were included in publications [18,19], where the effectiveness of fire retardants was between 75% and 88% measured with the MFT method [20]. Moreover, the effectiveness of chemical compounds is known and described in literature [21].

## 2. Materials and Methods

Samples with dimensions of 2 cm × 2 cm × 3 ± 0.2 cm were cut from Scots pine (*Pinus sylvestris* L.) sapwood boards. A total of 10 samples for each test variant were selected for the tests. The samples were selected according to sequence of occurrence in a given lath, without defects and visible changes caused by blue stain fungi [22,23]. The research variant consisted of a given chemical compound and the number of accelerated aging cycles. Each of the variants was tested separately. The test specimens were treated in vacuum dryers by the full-cell vacuum method using a vacuum of 0.1 MPa maintained for 20 min, and then the vacuum was gently reduced to atmospheric pressure and kept in solutions for another 2 h. After impregnation, the samples were air-conditioned for 14 days at the temperature of 23 ± 2 °C and humidity of 65 ± 5%, in a heated room with forced air circulation, until the wood moisture content was 10 ± 2%. The control samples had the same humidity throughout the test. The humidity of the samples was determined by the dryer-weight method [20]. The wood was treated with 6 solutions of chemical compounds with wood impregnation average gain (dry mass of chemical compound) (kg/m^3^): monoammonium phosphate (MAP) 62.85 kg/m^3^, boric acid (BA) 17.25 kg/m^3^, sodium tetraborate (borax) (Bx) 18.6 kg/m^3^, urea (U) 64.48 kg/m^3^, monoammonium sulphate (MAS) 66.51 kg/m^3^ and diammonium phosphate (DAP) 61.57 kg/m^3^, and a commercial preparation (FR) 59.98 kg/m^3^ at a concentration of 10%, except for borax and boric acid where the concentration was 4%. Tests were also carried out on wood not subjected to impregnation, i.e., control (C). Based on the literature, it was decided to develop a proprietary accelerated aging cycle simulating conditions in a temperate climate. An accelerated aging process was performed to determine progressive changes in the wood. The complete course of the aging process was 0, 8, and 16 cycles. Each cycle consisted of the following phases:

1. Heating (130 °C for 24 h)
2. Freezing (−15 °C for 24 h)
3. Heating (130 °C for 24 h)
4. Maintaining over a supersaturated solution of KNO_3_ giving approx. 90% (temp. 40–45 °C for 24 h)
5. Freezing (−15 °C for 24 h)

After the accelerated aging process, the samples were tested for physical changes and strength decline. Before the compression tests, samples were conditioned (until equilibrium moisture content (constant mass) was achieved) in the same conditions and mass as before the aging process, and their dimensions were measured using an analytical balance accurate to 0.001 g (Sartorius GmbH, Göttingen, Germany) and a digital caliper with accuracy to 0.01 mm [23]. Then, the beams were subjected on compressive strength test according to the ISO 13061-1:2014 [24], ISO 13061-5:2020 [25] and ISO 13061-17:2017 [26] standard on the Zwick Z100 testing machine (Zwick GmbH, Ulm, Germany) [23,27]. During the tests, the value of the compressive strength and Young’s modulus were recorded [28]. 

A statistical analysis of the obtained results was performed, starting with the determination of appropriate measures of central tendency (mean) and standard deviation (SD) [28]. The Kolmogorov–Smirnov test was used to verify the compliance with the theoretical normal distribution, and the homogeneity of variance was tested based on the Bartlett test. In order to determine the significance of the analyzed impregnation types and the number of aging cycles, a two-factor analysis of variance was used. Tukey’s HSD test was used to determine statistically homogeneous groups. The statistically significant results were those with *p* < 0.05. All calculations were performed in Statistica 13.3 software (StatSoft Polska Sp. z oo, Kraków, Poland).

## 3. Results

**Figure 1:**
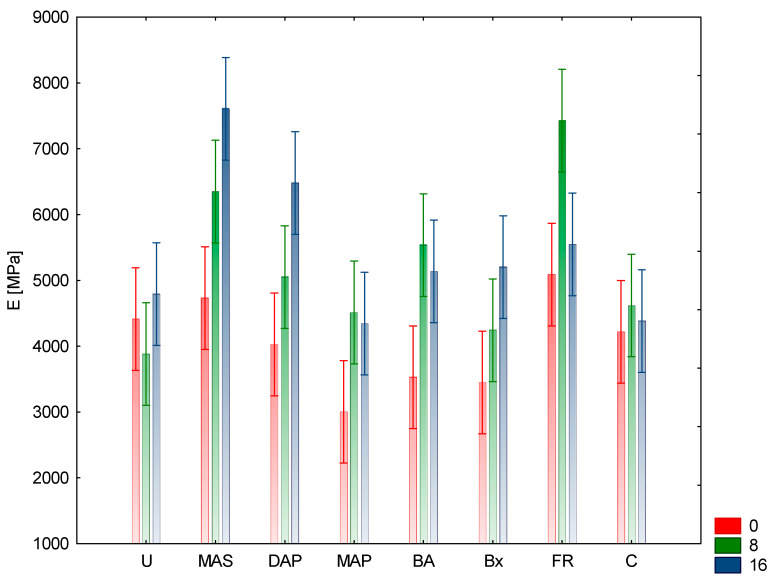
Illustration of the E relationship of aging cycles and chemical compounds. Whiskers represented 95% confidence interval.

**Figure 2:**
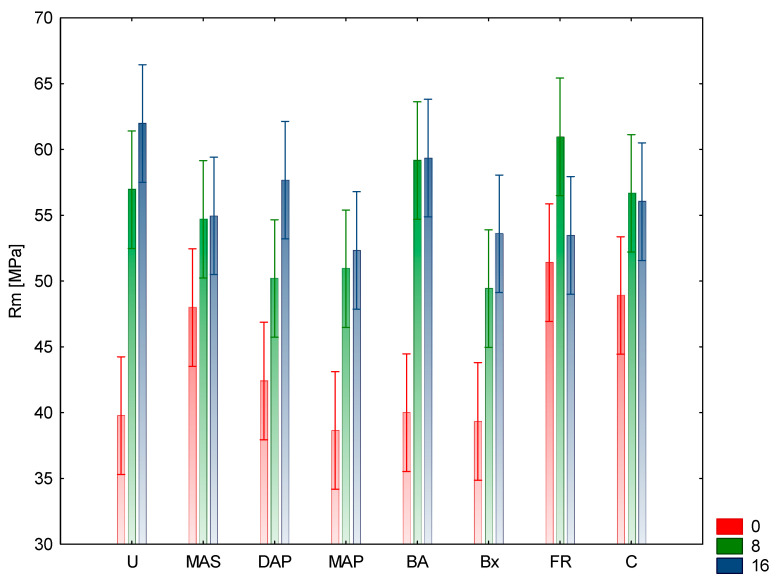
Illustration of the Rm relationship of aging cycles and chemical compounds. Whiskers represented 95% confidence interval.

The research results presented below reflect the average values of the analyzed features obtained for individual research variants. Analyzing the basic strength parameters, Rm (compressive strength) and E (Young’s modulus), it can be concluded that the highest value of compressive strength was obtained for samples protected with urea and subjected to the aging process for 16 cycles. In this case, Rm was 62 MPa. Samples protected with FR and aged for eight cycles were characterized by a slightly lower value (61 MPa). The lowest Rm values were observed for samples not aged, but protected with MAP, where the value was 38.7 MPa. Slightly higher values were obtained for samples protected with Bx and U, not subjected to aging. These values were 39.3 and 39.8 MPa, respectively. All the non-aged samples had lower Rm values compared to the samples protected with the same aged compounds (Figure 1 and Figure 2).

The absorption of the preparation and individual components was similar for the concentrations used. The obtained data show that the degree of absorption did not affect the strength properties of the protected wood. 

Analyzing changes in wood saturated with individual compounds, an increase in strength with aging time was found in the case of U, Bx, MAP, DAP, and MAS. For the above compounds, the increase in Rm over time from 0 to 16 cycles (assuming the values for the 0 aging cycle as reference) was, respectively, for the samples protected: with urea after eight cycles by 42.96% and after 16 cycles by 55.78%, with borax 25.7 and 36.39%, MAP 31.52 and 35.14%, DAP 18.39 and 36.08%, and MAS by 13.95 and 14.58%. For the remaining test variants, the increase in the strength value took place until the 8th aging cycle, while in the 16th cycle it was lower, however, the values did not reach the level for the 0 cycle. These values were respectively 15.95% and 14.52% for the control samples, 48.0% and 47.5% for the samples protected with boric acid, and 18.67% and 3.11% for the FR protected samples. Analyzing the above changes, it can be concluded that extending the aging process in the case of control samples and samples protected with boric acid does not significantly affect the changes in strength between the 8th and 16th cycle. The observed decrease in strength took place in the case of wood protected with FR, where the change amounted to 15.5%. This means that the compounds included in the composition of the preparation may act in an antagonistic manner, considering the influence on the compressive strength. The modulus of elasticity E showed similar dependencies in most of the analyzed research variants. A deviation from this relationship were the samples protected with urea, where the lowest mean value was obtained for samples aged for 8 cycles (Table 1). Significant increases in the value of E were observed after the 16th cycle of aging compared to the 0 and 8 cycles for compounds such as: Bx, MAS, and DAP. For wood treated with Bx, the increase in the value of E after the 8th cycle was 22.98% and after 16th cycle, 50.81% in relation to the 0th cycle as the reference aging. Similar values were obtained for MAS and DAP protected wood, respectively: 34.18% and 60.77% as well as 25.43% and 60.89%. In the case of control samples, the differences between the E values for the unaged and aged samples for 8 and 16 cycles were not that significant and amounted to 9.51% after the eighth cycle and 3.89% after the 16th cycle, respectively. For samples protected with BA and MAP, the drops in E values after the 16th cycle as compared to the 8 aging cycles were not that significant and amounted to 11.33% and 5.66%, respectively. The greatest changes in the values of Young’s modulus were observed for wood protected with FR, where after the 8th cycle of aging there was an increase by 45.96%, while after 16 cycles of aging only by 9.04% (the difference between the 8th and 16th cycles were 36.92%. Considering the composition of the FR preparation, it can be concluded that it does not adversely affect the strength properties of wood. Considering the obtained results concerning the basic strength parameters of the tested variants, it can be concluded that urea does not affect the values of the elasticity modulus E, regardless of the aging variant, as compared to the control samples. The highest positive effect on E values was demonstrated by MAS, which obtained higher E coefficient values with increasing time and number of aging phases. Moreover, samples protected with FR showed an increase in the E coefficient value during aging up to eight cycles. After 16 process cycles, the E value for FR dropped to a value similar to that of the unaged samples. In the case of the other variants, the E coefficient was lower than E for the control samples. In the case of DAP and Bx, the values of this coefficient after 16 aging cycles are higher than after eight and 0 cycles, while for samples protected with MAP and BA, after 16 aging cycles, the value of the E coefficient is lower than after eight. Such changes in the values of compressive strength and Young’s modulus compared to control wood, especially along with the elongation of the aging time, are caused by the chemical nature of individual components. The pH of the solutions of individual components was of the greatest importance with regard to the decrease in strength. The greatest changes were observed for the components whose pH was acidic (pH near 4–5): U, MAP, MAS, BA. The remaining compounds and the FRs had a pH close to neutral. Control samples without the aging process had a 5.3 pH value, measured using indirect cold water extraction method. Compounds with acidic pH cause, along with the prolongation of the action time, the hydrolysis of cellulose and hemicelluloses, thus lowering the strength properties of wood. Acidic fire retardants have the ability to catalyze the glucose dehydration process, resulting in cellulose depolymerization. This degrades the fibers, reducing their strength [29]. During the tests, no visual changes of the samples (cracks, twists, etc.) were found after the aging process. It was found that after the aging process, with the increase in the number of aging cycles, the weight loss of the protected samples increased. The highest weight loss was recorded for FR after 16 aging cycles and it amounted to 5.41% and 4.45% after eight cycles, and for MAS (4.18% and 2.95%, respectively). The lowest weight loss after aging was recorded for samples protected with Bx (2.1% and 1.28%, respectively) and BA (2.93% and 1.9%, respectively). The other variants showed weight loss in the range of 3.05–3.75% after 16 cycles and 1.7–2.21% after eight aging cycles. The control variants showed a weight loss after aging, regardless of the number of cycles, at the level of 0.75%. These results may indicate the hydrolytic activity of individual components of the preparation.

In the case of the feature of the Young’s coefficient (E), both the relationship (*p* = 0.000) and the number of aging cycles (*p* = 0.000), as well as their interaction (*p* = 0.000), show a statistically significant influence on the difference in its mean values for individual research variants [30]. Based on the results of Tukey’s post-hoc test, it can be concluded that the eight and 16 cycle variants show statistically similar results, while the 0 cycle aging variant differs from the other variants. In the case of chemical compounds, due to the values of the E factor, four homogeneous groups can be distinguished (Table 1).

For all non-aging variants except FR, the Rm values are lower than for the control variant. Moreover, the variants aged for eight cycles show Rm values lower than those of the control variant. The exceptions are variants protected with boric acid, FR and urea. For the variants aged for 16 cycles, the Rm values for the samples protected with boric acid and urea were also higher than for the control variant.

Inference similar to the E feature was carried out for the Rm feature. Again, the aging (*p* = 0.000) and the relationship (*p*= 0.000) as well as their interaction (*p* = 0.001) turned out to be statistically significant. As for the aging effect, again, eight and 16 cycles give similar results, and the aging variant 0 differs from them. In the case of Rm values, the homogeneous groups are arranged differently than for E due to the compounds (Table 1).

**Figure 3:**
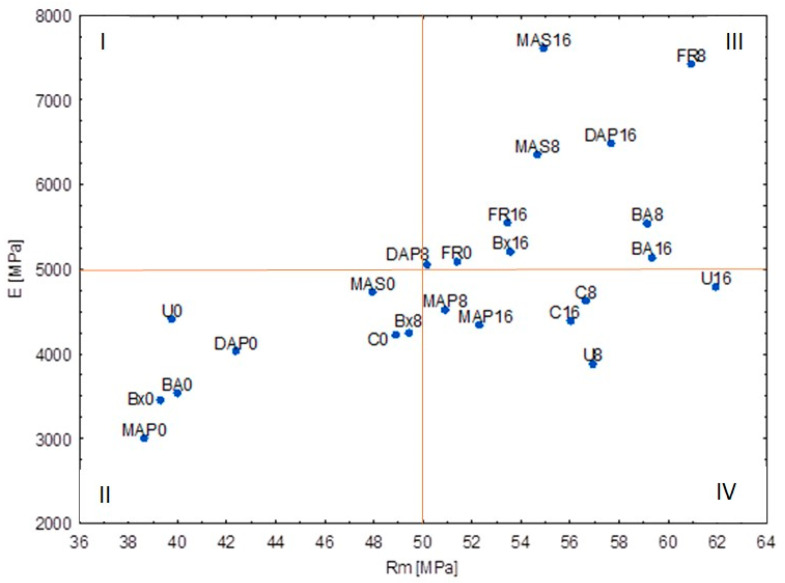
Illustration of the Rm and E relationship of aging cycles and chemical compounds. I–IV are sections of graph.

Figure 3 shows the simultaneous levels of two features (E and Rm) depending on the combination compound × aging. This represents an illustration of the interaction of both factors on the two considered strength characteristics.

Analyzing the relationship between the chemical compounds, aging cycles, and their influence on the strength parameters (E and Rm), it can be stated that the compounds in the Section III of the graph have a positive effect on these parameters (in the studied period), and the negative ones in the Section II. Variants close to the center can be considered the most neutral in terms of strength changes in relation to the controls without aging process.

Applying the analysis of variants, considering at the same time the average values of the observed properties (E and Rm), it can be concluded that the best strength properties were shown by such compounds with aging cycles as FR 8, MAS 8 and 16, and DAP 16. This is confirmation supporting a positive effect on the strength properties of wood in an aging time context. The worst effects were demonstrated for the variants not subjected to aging, i.e., those protected with MAP, DAP, Bx, BA, and U. Moreover, these compounds, in the variant without aging, showed the most negative impact on the strength properties of wood.

## 4. Conclusions

Extending the aging process in the case of control and boric acid-protected samples does not significantly change the strength between the eight and 16th cycles. The greatest changes in strength were shown by FR.

Some of the compounds contained in FR have an antagonistic effect related to the compressive strength of wood. The greatest influence on this phenomenon is probably the content of boric acid and MAP. Due to the lack of detailed data on the chemical composition of the preparation, it is not possible to clearly determine which of these compounds causes the greatest changes. This is indicated by the results obtained during laboratory tests and their statistical analysis. Changes in the compressive strength and Young’s modulus compared to control wood, especially along with the elongation of the aging time, are caused by the chemical nature of individual components. The pH of the solutions of individual components was 321, which is of the greatest importance with regard to the decrease in strength. Acidic FRs can lead to the glucose dehydration process, resulting in cellulose depolymerization and reducing strength of wood fibers.

## Figures and Tables

**Table 1:** Summary of statistically homogeneous groups determined on the basis of the HSD Tukey test for E and Rm. The symbol * meaning that results are not statistically different.

Solution	E-Mean	1	2	3	4	Rm [MPa] Mean	1	2
MAP	3953.00	*				47.30	*	
Bx	4298.67	*	*			47.45	*	
U	4361.33	*	*			50.09	*	*
C	4406.33	*	*			52.54	*	*
BA	4733.33	*	*			52.84	*	*
DAP	5185.67		*	*		52.89	*	*
FR	6019.67			*	*	53.86		*
MAS	6228.33				*	55.28		*

## Data Availability

Datasets are available in corresponding author archive.
